# CALORIC RESTRICTION AND AGING: FROM CALORIES TO FASTING Times

**DOI:** 10.1093/geroni/igad104.1357

**Published:** 2023-12-21

**Authors:** Rafael de Cabo

**Affiliations:** National Institute on Aging, National Institutes of Health, Baltimore, Maryland, United States

## Abstract

The caloric content and manipulations of nutrient composition have defined our research focus over decades, leading to the formulation of ideal diets and supplements for every phase of life. In the last few years, the control of meal size, frequency, and timing have become a powerful strategy to ameliorate and postpone disease onset and delay aging. Emerging evidence highlights the importance of prolonged fasting periods for the health and survival benefits of calorie restriction (CR) and time-restricted feeding (TRF) across multiple species. Furthermore, there is new evidence of the potential combinatorial use of these dietary interventions with established therapeutical approaches to enhance outcomes, particularly in anticancer treatments.

